# Sensing nitriles with THz spectroscopy of urine vapours from cancers patients subject to chemotherapy

**DOI:** 10.1038/s41598-022-22783-z

**Published:** 2022-10-27

**Authors:** Vladimir Vaks, Vladimir Anfertev, Maria Chernyaeva, Elena Domracheva, Anton Yablokov, Anna Maslennikova, Alla Zhelesnyak, Alexei Baranov, Yuliia Schevchenko, Mauro Fernandes Pereira

**Affiliations:** 1 Institute for Physics of Microstructures, Nizhny Novgorod, 603950 Russia; 2 Lobachevsky State University, Nizhny Novgorod, 603950 Russia; 3 Privolzhsky Research Medical University, Nizhny Novgorod, 603005 Russia; 4 Nizhny Novgorod Regional Oncology Hospital, Nizhny Novgorod, 603000 Russia; 5 Institute of Electronics and Systems (IES), University of Montpellier, UMR5214 CNRS/Université, Montpellier 2, 34095 Montpellier, France; 6 Department of Physics, Khalifa University of Science and Technology, 127788 Abu Dhabi, United Arab Emirates; 7 Institute of Physics, Czech Academy of Sciences, 18221 Prague, Czech Republic

**Keywords:** Photonic devices, Chemotherapy

## Abstract

A THz nonstationary high-resolution spectrometer based on semiconductor superlattice multipliers is applied to investigate the dynamics of urine composition for cancer patients treated with chemotherapy. The molecular urine composition of healthy volunteers and cancer patients was compared and contrasted. We have found a set of nitriles that either appeared after chemotherapy or increased in content, which are expected as a result of bio-chemical damage to the liver. While no damage can be detected at this stage by existing clinical methods, the identified nitriles are candidates for further large-scale systematic testing towards markers for nephrotoxicity of chemotherapy at an early stage of the treatment, when conventional diagnostics cannot identify substantial organ damage. Comparing the metabolite concentration dynamics with side effects during chemotherapy might then help individuate patients prone to severe complications and correct the treatment. Our devices are game-changers for THz spectroscopy of liquids: they allow spanning four different frequency ranges for a general evaluation of most substances found in the liquid and selecting a spectral interval that bypasses the strong absorption lines from substances such as water and ammonia, which may otherwise mask the detection of the target metabolites.

## Introduction

Terahertz (THz) photonics is developing at large speed with the increasing availability of new materials, sources, and detectors^1–5^, and we focus here on what may become one of its most impactful applications: Metabolomics, which is the systematic study of the chemical compounds—metabolites, stemming from cell metabolism.

Metabolites and their concentrations are directly connected to the underlying biochemical activity and state of cells, tissues and organs, providing an opportunity for the development of new diagnostic techniques. In particular, the analysis of the chemical composition of exhaled breath and biological liquids (blood, saliva, urine) has been proven to provide important information about diseases and pathological processes in organisms^6^.

This very timely study aligns with the significant focus on awareness of comorbidities following the increase in cancer survival rates after chemotherapy^7^. Here we directly monitor early-stage metabolic alterations due to chemotherapy with our THz spectroscopy technique^8^.

The toxic influence of chemotherapy ranges from subclinical manifestations, which have no influence on the quality of life, to life-threatening states^9^. Any exogenous or endogenous organism intoxication leads to an increase in the concentration of chemical compounds, mostly the intermediate products of peroxidation, such as malondialdehyde, conjugated dienes and trienes, aldehydes and ketone groups of protein carbonyl derivatives, dityrosine, tryptophan, protein thiol groups and others^9^. Detection of the intoxication products after the initiation of a course of chemotherapy may reveal minor dysfunctions of metabolism^7^, which cannot be detected with standard clinical techniques. Comparison of the metabolite’s concentration dynamics with side-effects, appearing during the course will help to identify patients prone to serious complications and correct the treatment.

Existing techniques used in clinics for monitoring metabolites are limited to standard laboratory methods, such as biochemical blood tests and clinical urine analysis. However, they can only reveal significant organ failure. It is thus of relevance to develop techniques that indicate subtle side effects that already start at the beginning of chemotherapy, which cannot be detected by the current standard laboratory tests.

In the case of renal failure caused by platinum-based chemotherapy, there are, for example, changes in serum creatinine levels and creatinine clearance. Note, however, that clinically detectable nephropathy (changes in biochemical parameters) as a result of chemotherapy very rarely occurs after 1–2 courses of nephrotoxic chemotherapy. As a rule, changes in biochemical parameters appear after 4–6 courses of chemotherapy, i.e., 4–6 months after the start of the treatment. The aim of our work is to build a sensor, be able to interpret the resulting data and find candidate markers that might be correlated to subtle damage to the kidney tissue immediately after exposure to cisplatin before the appearance of other measurable clinical symptoms. In other words, we wish to determine guidelines to detect the onset of damage before it develops into damage to the organ. Suitable large scale studies will follow.

The powerful approach to studying multi-component mixtures described here is non-stationary high-resolution THz spectroscopy^10,11^. The method can be applied to gases, vapors of liquids (such as urine and blood), or solid-state samples, obtained after natural evaporation or thermal decomposition. The advantages of this technique in the analysis of biological samples include a wide range of detectable compounds and their unique identification, real-time measurements, the use of easy-to-obtain samples, and suitability for any patient regardless of their state.

The THz spectrometer developed for this study is based on semiconductor Superlattice Multipliers (SSLMs) with input taken from commercial sources: a Gunn diode and a Backward Oscillator (BWO)^12–14^. Superlattices are very efficient for frequency multiplication from sub-THz up to the THz range, but they require complex gold resonators both for emitter and detector, which limits frequency tuning, increases the cost of the device, and reduces the output power^11,14^. There is thus a large potential for further improvement of these devices. One possible improvement would be on-chip integration with superlattice electron devices. These room-temperature compact sources can already deliver 4.2 mW power output at 145 GHz^15^. If we further synchronize the SSMLs, a significant increase in output power can be achieved^16,17^.

Our recent success in orders of magnitude control of the nonlinear mechanisms responsible for frequency multiplication in SSLMs^18–25^ helped us control the input power to optimize the spectrometer to monitor the vapor composition of urine from cancer patients, undergoing platinum-based chemotherapy. Before moving forward, we should mention that quantum cascade lasers are efficient mid-infrared sources, but do not operate in the THz range without cryocooling and cannot reach the low THz range covered in this study^26–29^.

## Results and discussion

Our pilot study was carried out on six patients who underwent platinum-based chemotherapy with permission from the Local Ethical Committee of Nizhny Novgorod Regional Oncology Hospital, Nizhny Novgorod, Russian Federation. The patient’s characteristics are given in the section: “Materials and methods” in Table 2. This study is not about cancer itself, but actually about the possible damage caused by chemotherapy with platinum derivatives, since nephrotoxicity is the limiting side effect of treatment when using this class of cytostatics. Damage to the renal parenchyma with the development of renal failure of varying severity can cause interruption or cancellation of treatment in this category of patients, so it is crucial to monitor its onset. Our study included patients with the following tumors: oral cancer, oropharyngeal cancer, lung cancer, and ovarian cancer. According to clinical guidelines^30^, chemotherapy with platinum derivatives is the standard treatment for all these cancers. All patients received chemotherapy with platinum drugs, so we can expect them to develop renal toxicity regardless of which other drugs are included in the treatment (paclitaxel, etoposide, 5-fluorouracil). To guarantee that the appearance of markers that might be correlated to subtle damage to the kidney tissue after chemotherapy, it is important that all participants in the study had no renal insufficiency. The urine and blood tests with normal indices confirmed the status of the kidneys of both healthy volunteers and cancer patients. Thus, all participants before chemotherapy plus the healthy patients make up a control group for our study.

Our SSLMs deliver enough power to multiply the fundamental source frequencies in the 118–175 GHz range to 3rd, 5th and 7th harmonics. This allow us to search for signatures of the candidate biomarkers (nitriles) expected to appear in the urine of patients undergoing chemotherapy at an early stage. This is not an easy task, because in all spectral ranges, notably on the 3rd, 5th and 7th harmonic ranges, there are other substances mixed up, such as ammonia (NH_3_) and water (H_2_O). Supplementary Tables S1 to S3 show spectral data of representative examples of substances found in all samples (Table S1), typical substances measured in the urine sample of patient 2 after chemotherapy (Table S2) and some of the nitriles measured in the urine sample of patient 2 after chemotherapy (Table S3). The Supplementary Tables also illustrate how we identify the substances found in the urine vapor by comparing experimental lines with corresponding data found in the JPL and Köln databases^31,32^.

Doppler broadening, characteristic of the absorption lines, is proportional to frequency, which combined with many strong lines of interfering substances, which are detected as one broadened line higher, make the detection of target substances difficult in the higher harmonic ranges. We have thus completed the study using the BWO-based spectrometer in the 2 mm range (118–175 GHz). There is only one ammonia absorption line and the nearest absorption line for water is at f_H2O_ = 183.310087 GHz, outside this range. The possibility of analyzing four different frequency ranges to confirm the presence of target compounds, including a water absorption-free range, not accessible by usual THz sources, such as quantum cascade lasers^26–29^, make our method a unique tool to investigate liquids.

**Figure 1:**
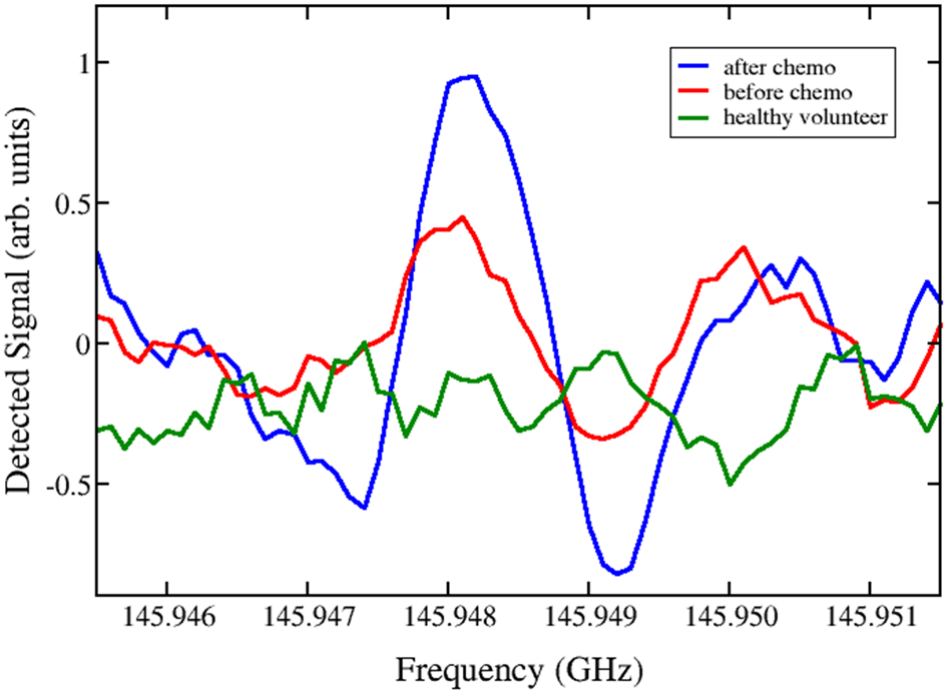
Experimental detection of the absorption spectral line of isobutyronitrile (i-C3H7CN) near the frequency f_c_ = 145.9481 GHz, measured in the sample of patient 2 before (solid, red) and after chemotherapy (solid, blue), together with the corresponding reference measurement for healthy volunteer 1 (dashed, green). There are two close lines near this frequency at f_−_ = 145.9478961 GHz, characterized in the databases^31,32^ by Lg I = − 5.0754 and quantum numbers 38 5 33 ← 38 5 34 (v30 = 1) and f_+_ = 145.9479272 GHz, with Lg I = − 5.0754 and quantum numbers 38 6 33 ← 38 4 34 (v30 = 1). These two lines overlap and are detected as one.

**Figure 2:**
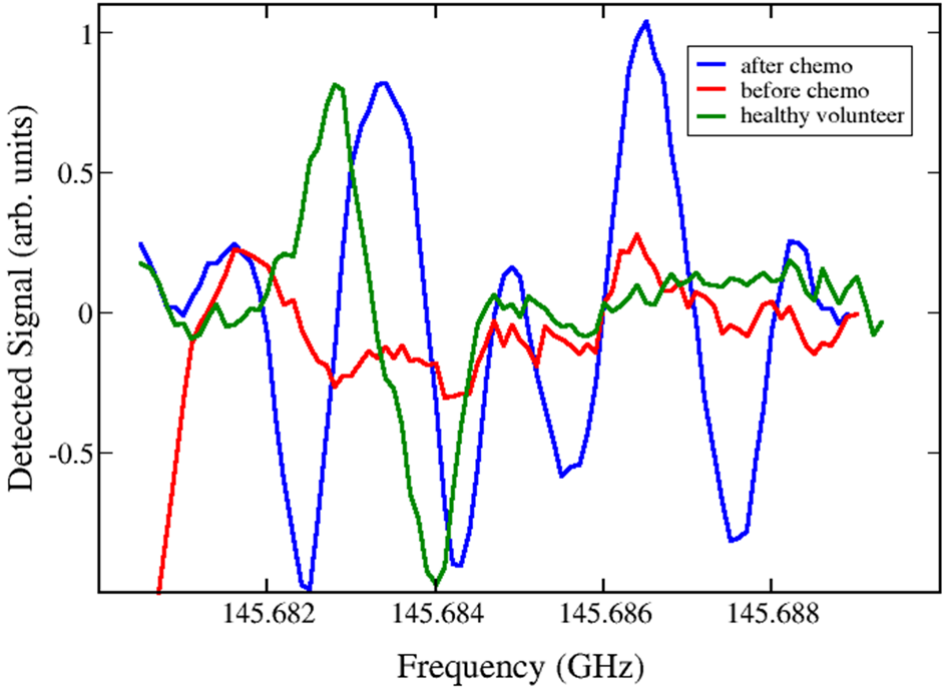
Experimental detection the absorption spectral lines of methyl butyronitrile (C_2_H_5_CHCNCH_3_) near the frequency f_c1_ = 145.6832 GHz and glycine conformer II (H_2_NCH_2_COOH) near the frequency f_c2_ = 145.6864 GHz detected in the sample of patient 2 before (red) and after chemotherapy (blue), together with the corresponding reference measurement for healthy volunteer 1 (green). There are two close lines differing by less than tens of Hz for methyl butyronitrile, given in the databases^31,32^ at the frequency fc = 145.6833846 GHz, characterized by Lg I = − 4.9041 and quantum numbers (v = 0) 64 54 10 ← 64 53 11 and (v = 0) 64 54 11 ← 64 53 12. For glycine conformer II, there is only one line at the central frequency fc = 145.6860831 GHz, characterized by Lg I = − 3.1037 and quantum numbers 21 4 18 ← 20 4 17 in the databases^31,32^.

**Figure 3:**
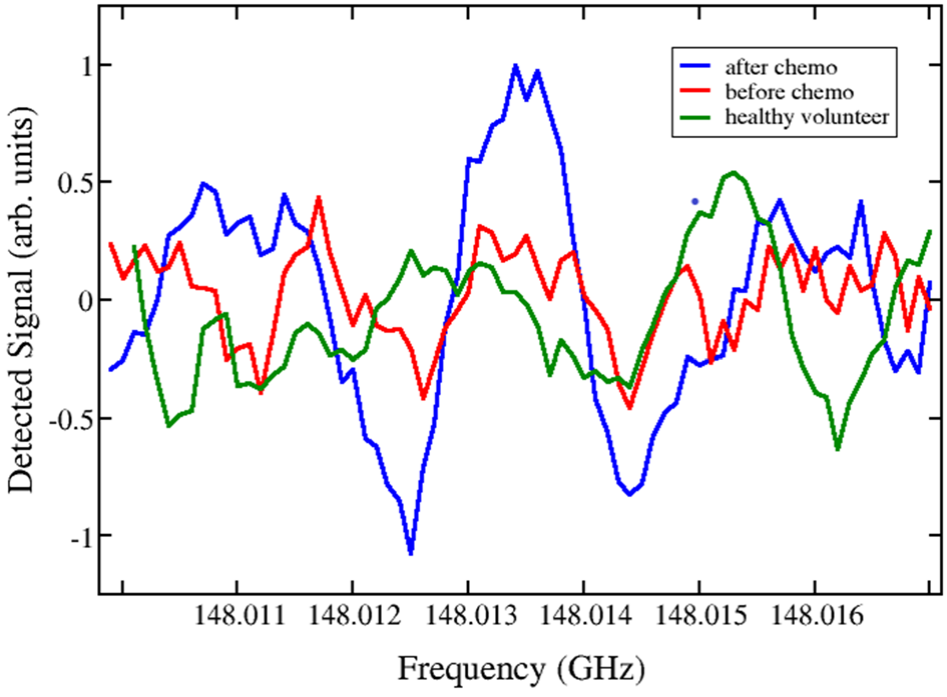
Experimental detection of the absorption spectral line of ethanethiol (a-C2H5SH) measured in the sample of patient 2 before (solid, red) and after chemotherapy (solid, blue), compared with the corresponding reference measurement for healthy volunteer 1 (dashed, green). There are two quantum transitions near this frequency at f = 148.0132725 GHz, characterized in the databases^31,32^ by Lg I = − 5.5024 and quantum numbers 45 14 31 ← 46 13 34 and 45 14 32 ← 46 13 33.

Before proceeding further, we highlight that even though urine is heated up to about 200 °C, the vapors enter the measuring cell at room temperature and the absorption line shape depends on temperature and vapor pressure. We have large spectral broadening and detect small concentrations of nitriles, which are mixed with other substances such as ammonia (NH_3_) that have much stronger spectral lines in the spectral ranges that we study. As a consequence of these factors, the signal-to-noise-ratio (SNR) for the detected nitriles is about SNR = 3:1. The SNR in absorption lines can be conclusively resolved for SNR about 3 or lower and there are methods allowing to resolve lines for even smaller SNR, close to 1. In our case, the defining factor is not of good or bad SNR, but rather weather a line that has a spectral position found in JPL and Köln databases, with specific form and linewidth, is either present in the spectrum or not. The absorption lines, which are detected in the phase-switching mode characteristic of our sensor, are quasi second derivatives from Voight line shapes. A detailed discussion for cases like ours is given in Ref.^45^. We have measured too many spectral lines and showing all of them would make the paper unreadable. We show three representative absorption lines in Figs. 1, 2 and 3, for of isobutyronitrile (i-C3H7CN), butyronitrile (C2H5CHCNCH3) and ethanethiol (a-C_2_H_5_SH), respectively, measured in the sample of patient 2 before (solid, red) and after chemotherapy (solid, blue), compared with the corresponding reference measurement for healthy volunteer 1 (dashed, green). The quantum number convention and the definition of intensities with Lg I are standard in molecular physics and are reviewed in detail in the Supplementary Material.

Table 1 summarizes the results of spectroscopic studies of the urine from healthy volunteers (HV1, HV2) and cancer patients before (BC) and after chemotherapy (AC).

**Table 1:** Vapor composition of the urine, taken from healthy volunteers (HV1, HV2) and cancer patients before (BC) and after chemotherapy (AC).

Substance	Patient													
	1		2		3		4		5		6		HV1	HV2
	BC	AC	BC	AC	BC	AC	BC	AC	BC	AC	BC	AC		
	Number of substance absorption lines in the sample spectrum													
Isocyanic acid	4	9	6	10	12	10	11	16	15	14	14	14	15	14
Ammonia	1	1	1	1	1	1	1	1	1	1	1	1	1	1
Ammonia with deuterium	0	1	0	0	2	2	0	1	1	0	1	1	0	1
Propionitrile, including isotopologues with deuterium and C13	1	0	0	2	0	0	0	0	0	0	1	3	0	1
Butironitrile, including isotopologue with C13 and isobutyronitrile	1	3	1	3	2	1	3	2	2	4	2	6	2	1
Pentannitrile	0	3	1	2	0	3	2	5	2	2	3	5	2	0
Methylbutironitrile	1	3	1	5	1	0	0	3	0	0	3	6	0	0
Acetonitrile	0	5	0	0	4	4	0	0	0	0	0	0	0	0
Hydroxyacetonitrile	0	0	3	2	0	0	0	0	0	0	0	1	0	0
Methyleneaminoacetonitrile	0	2	0	0	1	0	0	0	0	0	2	2	0	0
Mercaptoacetonitrile	1	1	0	1	1	0	0	0	0	0	0	3	0	0
Aminopropionitrile	1	2	0	0	0	0	0	5	0	3	4	3	0	0
Acrylonitrile, including isotopologue with c13	1	1	0	2	0	0	0	0	0	0	0	3	0	0
Oxomalone nitrile	0	0	1	4	1	1	0	0	0	0	0	0	0	0
Ethanethiol	0	1	0	1	0	1	0	2	0	0	0	0	0	1
**Propionitrile with one or more C isotopes**	**0**	**1**	**0**	**2**	**0**	**1**	**0**	**1**	**1**	**2**	**0**	**1**	**0**	**0**
**Acrylonitrile including isotopologue with deuterium**	**0**	**0**	**0**	**1**	**0**	**0**	**0**	**0**	**0**	**0**	**0**	**0**	**0**	**0**
**Aminoacetonitrile with C isotope**	**0**	**0**	**0**	**1**	**0**	**0**	**0**	**0**	**0**	**0**	**0**	**0**	**0**	**0**
**Aminoacetonitrile, including isotopologue with deuterium**	**0**	**0**	**0**	**2**	**0**	**0**	**0**	**1**	**0**	**0**	**1**	**3**	**0**	**0**
**Benzonitrile**	**0**	**3**	**0**	**2**	**0**	**0**	**1**	**3**	**0**	**3**	**2**	**5**	**0**	**0**

The candidates for markers of subtle renal damage are marked in bold.

In previous investigations^33–35^, the reactions of thermal decomposition of the urea were detected upon heating the samples of other biological liquids such as capillary blood and blood plasma. Isocyanic acid and ammonia can appear in the gas mixture of vapors of urine samples as products of thermal decomposition of the urea during heating up to temperatures higher than its boiling point, 174 °C. However, ammonia and isocyanic acid were present in the urine vapors also before heating. Therefore, these compounds may originate from another source. For example, proteins, consumed with food, are naturally decomposed into amino acids and subsequently degraded into various products, such as ammonia^36^.

It is a complex task to find a common peak in all chemotherapy patients that appears after treatment that was not there before since there are many substances whose content and quantity of absorption lines correspondingly change (increase or decrease) after chemotherapy. As matter of fact, the spectral analysis allows to classify the volatile compounds detected into 4 groups: (i) Substances found in all samples of both healthy volunteers and cancer patients: water, deuterated water, ammonia and deuterated ammonia, isocyanic acid, urea, formic acid, acetic acid, formamide, and butyronitrile. Deuterated compounds in urine can be formed from deuterated water, contained in normal water at a concentration of one molecule of HDO per 3200—3800 molecules of H2O. (ii) Substances that were detected after chemotherapy, but also in one of the healthy volunteer samples, such as Acetaldehyde, propionitrile, including isotopologues with deuterium and C13, pentannitrile and ethanethiol. (iii) Nitriles that do not appear in healthy patients’ samples and mostly appear after chemotherapy or have an increased number of lines in average, but show some unusual behavior, such as disappearing after treatment for some of the patients: methylbutironitrile, hydroxyacetonitrile, methyleneaminoacetonitrile, mercaptoacetonitrile, aminopropionitrile, or being present before treatment but not changing for some cases such as oxomalonenitrile acrylonitrile, including the isotopologue with C13 and acetonitrile. (iv) Nitriles that were not found in healthy patients’ samples, but either appeared in the urine of patients exposed to chemotherapy or had an increased number of existing lines, namely: benzonitrile, aminoacetonitrile, including the isotopologue with deuterium, aminoacetonitrile with C isotope, acrylonitrile including the isotopologue with deuterium, propionitrile with one or more C isotopes.

Even though a large scale study of all nitriles that appear is needed, group (iv) nitriles are our marker candidates for future large scale investigations that will follow many patients through the whole treatment with a deep statistical analysis. There was no other additional treatment and no deviation from the usual diet. Thus the increase in group (iv) nitriles after chemotherapy cannot be the product of the metabolization of any other drug or food.

It is important at this point to summarize relevant information about the relationship between metabolites found in this study and chemotherapeutic drug-metabolizing. For a review, see Ref.^37^. Cell injury takes place after cisplatin is converted to nephrotoxic molecules in the proximal tubule^37^. The highest concentration of cisplatin is found in cytosol, mitochondria, nuclei, and microsomes^38^. Cisplatin is conjugated to glutathione and then metabolized through glutamyl transpeptidase and cysteine S-conjugate-lyase–dependent pathways to a reactive thiol, which is a potent nephrotoxin. Glutamyl transpeptidase is located on the cell surface, whereas cysteine-S-conjugate-lyase is an intracellular enzyme. Inhibition of these 2 enzymes has no effect on the uptake of cisplatin into the kidney but reduces nephrotoxicity. Inhibition of glutamyl transpeptidase activity, however, renders cisplatin inactive as an antitumor drug. Whether inhibition of cysteine S-conjugate-lyase affects the antitumor activity of cisplatin is not known^37,39^. One of the thiols cited above, i.e. ethanthiol was detected in the content of urine in sample 2 after chemotherapy and the corresponding spectra are shown in Fig. 3.

One of the possible candidates among proteins, containing cysteine^40^ and present in urine, is the Tamm-Horsfall protein (THP). It appears in urine as the proteinuria^41^. Each subunit of THP contains about 50 cysteine residues, which form disulfide bonds^42^. THP was shown to have an immunomodulatory influence on the immune cells. Furthermore, THP can be used as a biomarker for chronic and acute kidney disease^43^. Hence, the presence of the nitriles in urine vapors may arise from the decomposition of THP and, thus, be an indicator of subclinical renal toxicity, which cannot be detected by standard clinical methods.

Moreover, nitriles are not readily metabolized and will remain largely unchanged in the body, meaning that their detection in the urine suggests toxicity and the potential of our diagnostic method. However, there is currently no information that the early subtle treatment toxicity can somehow affect the course of the tumor process and the prognosis of patients.

## Conclusions and outlook

A high-resolution THz spectrometer based on Gunn generators and semiconductor superlattice multipliers and mixers was employed to analyze the composition of urine vapors from cancer patients, undergoing nephrotoxic chemotherapy, and conditionally healthy volunteers, representing a control group. The study did not involve patients that had already developed cisplatin-induced nephropathy.

Nitriles are expected as a consequence of biochemical reactions associated with kidney damage due to chemotherapy. We have indeed found several nitriles, that cannot be detected at this stage by existing clinical methods and which we classified in 4 groups. The group (iv) are nitriles that either appeared after chemotherapy or increased their content are our initial candidates for large scale studies to follow patients throughout the treatment with a deep statistical analysis, to conclusively correlate the onset of nitriles detection with liver damage. Our goal here was to develop a dedicated spectrometer, measurement technique and an initial set of candidate markers as starting point for such large scale studies.

A game-changer in the technical development of gas analysis was the application of semiconductor superlattice structures in the THz gas nonstationary spectrometer applied for urine analysis. The combination of a cryogenic trap, reducing the amount of water, plus detection in 4 distinct frequency ranges, with one of the frequency intervals free of water and other interfering absorption lines, makes our method unique for studying targets in solutions that cannot otherwise be studied with the usual THz sources. The results of the studies demonstrate that mixers and multipliers, based on these structures, can significantly improve the characteristics of the spectrometer for high-precision analysis of biological gases and liquids, raising metabolomics to a much higher level of relevance in medicine and paving the way for a long-term systematic study of current and the novel treatments of diseases and possible toxic effects that they can impart on the organism. This work further opens the possibility of analyzing a plethora of other liquids that, so far, cannot be accessed by existing THz sources and detectors.

## Materials and methods

### Spectrometer based on semiconductor technology

Prototype THz high-resolution spectrometers based on SSLMs for the analysis of multicomponent gas mixtures have been developed and used in various applications, including medical diagnostics. Backward Wave Oscillators (BWOs) and Gunn Generators have been used to provide input radiation for both the excitation source and the heterodyne detection scheme and are described in detail in Ref.^11^. However, in contrast to Ref.^11^, here the frequency of the reference generator for the phase-lock loop (PLL) system is 260 MHz and 200 MHz for the radiation source and the oscillator of the heterodyne receiver, respectively.

In the clinical analysis described in this paper, we used 3rd to 7th harmonics of the radiation output of a Gunn generator. The frequency of the Gunn generator in the heterodyne receiver is stabilized using the PLL system at the reference frequency synthesizer. The SSLM delivers the intermediate frequency (IF) signal to the heterodyne receiver. The IF signals are formed between the frequency of a signal detected and the heterodyne signal frequency divided by a difference between the frequencies of the radiation source and heterodyne harmonics: f_IF_ = M*60 MHz, where M = 3 ÷ 7. The IF frequency ranges from 180 to 420 MHz, respectively. The frequency multiplier is built on a 11.2-nm-thick GaAs/AlAs superlattice made up of 18 repetitions of GaAs and AlAs layers (5.1 nm and 1.1 nm, respectively). The superlattice was homogeneously doped with silicon to 2 × 10^18^ cm^−3^ and enclosed between Si-doped GaAs contact layers doped to 2 × 10^18^ cm^−3^. To avoid undesired potential drops, the superlattice was separated from the contact layers by 32-nm-thick graded GaAs/AlAs regions where the AlAs to GaAs ratio gradually decreased from 1/6 to 1/28. The wafer was grown by molecular beam epitaxy in a RIBER 412 solid source reactor on an undoped (001) GaAs substrate. The grown wafer was processed into 1 × 1 µm^2^ deep mesa devices using conventional UV photolithography. The top AuGe-Au contact with the active mesa was formed by an air bridge. The opposite side of the active mesa was connected to the large area anode of the device through the bottom contact layer and the whole structure. The fabricated SL multiplier was placed in a waveguide chamber to provide efficient impedance matching with BWO or Gunn Generator radiation. The power supplied to the frequency multiplier input varied from 5 to 10 mW.

Previous studies have demonstrated harmonics from a BWO generator with a reference frequency of 150 GHz, up to 8.1 THz, corresponding to the 54th harmonic^11^. However, the power of these harmonics was not sufficient to perform spectroscopic measurements. Therefore, optimization and further study of the SL structures are required to achieve higher output power and a lower level of noise.

Optimization is achieved in a joint effort combining predictive simulations and experiments. The output of the SSLMs is independent of the type of source used to generate input frequency, and both the THz source and heterodyne detection scheme are successfully described by a predictive simulation scheme, that is based on Nonequilibrium Green’s Functions (NEGF) calculations and analytical solutions for the Boltzmann equation, as described in detail in the literature^18–25^. It is also possible to extract the relevant parameters from experimental current–voltage curves.

**Figure 4:**
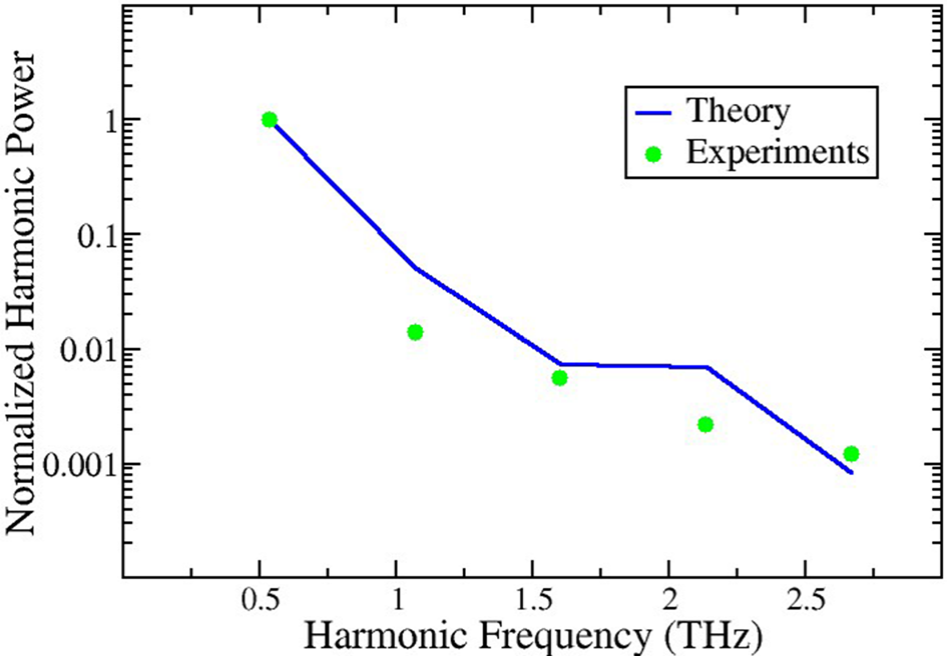
Normalized output power ${P}_{n}/{P}_{3}$ for the 3rd, 6th, 9th, 12th and 15th harmonics. The input frequency is $\nu =$ 178 GHz and $\alpha =eEd/h\nu =$ 19.6. Using the vacuum impedance and frequencies in GHz the connection between the $\alpha$ parameter that leads to power control of the multiplication and the plane wave power in mW is ${P}_{in}\left(\mathrm{mW}\right)=29.6\times {10}^{-10}{\nu }^{2}{\alpha }^{2}.$ The experimental data are represented by (green) symbols, and the solid (blue) line is calculated using Refs.^18–25^. Input parameters are obtained from NEGF calculations for the current–voltage using the nominal parameters for the sample, as provided by the sample grower.

**Figure 5:**
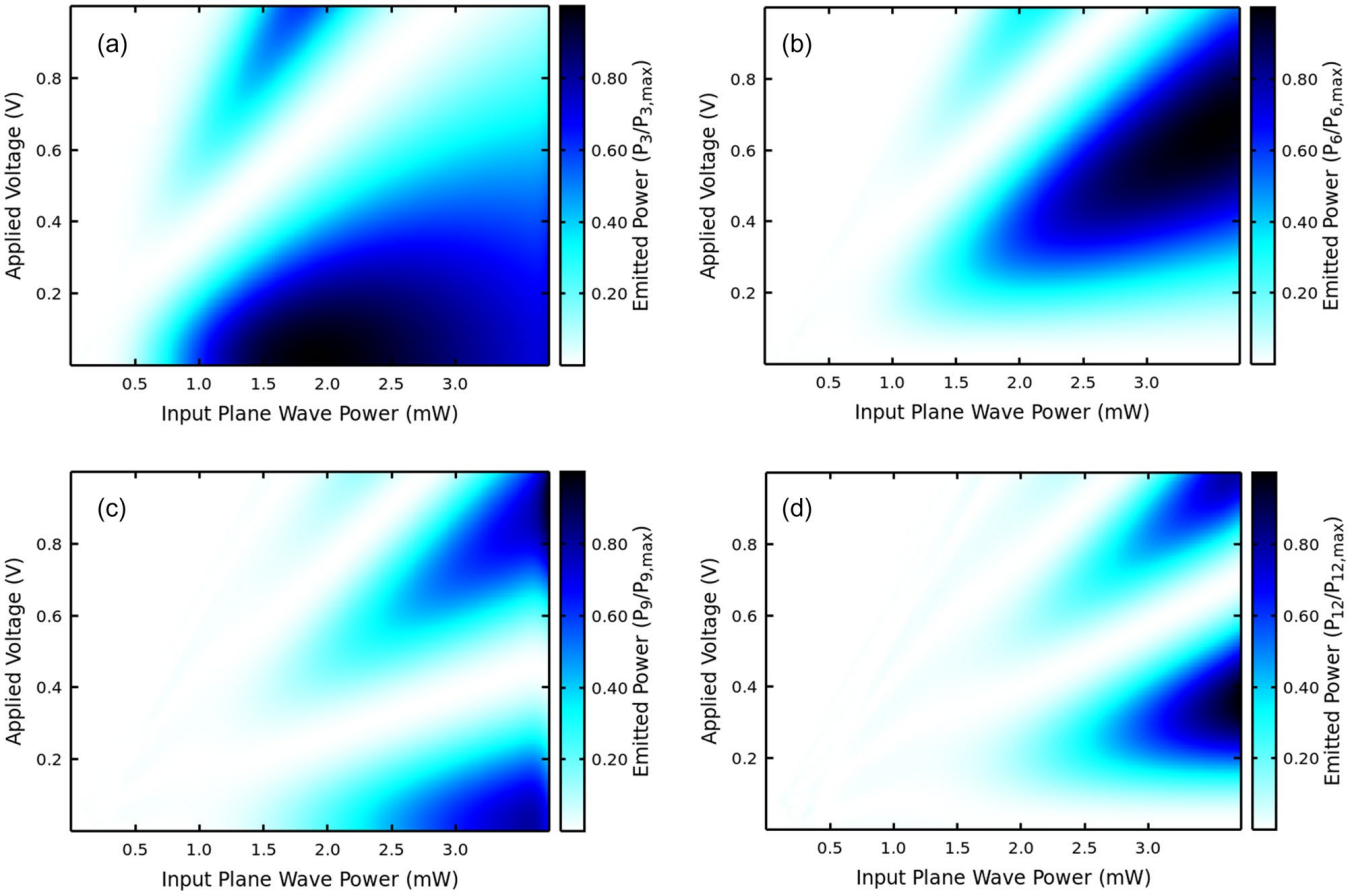
Emitted harmonic powers as a function of plane wave power directly transmitted to the GaAs-AlAs SSLM and applied voltage. Each figure is independently normalized for easier visualization since, as shown in Fig. 4, there is a 3 order of magnitude drop in output from the 3rd to the 27th harmonic. From (**a**–**d**) the figures correspond to the 3rd, 6th, 9th and 12th harmonics of the input frequency at 178 GHz, i.e.: 534, 712, 890 and 1068 GHz. The power in the x-axis is calculated using the assumption of a plane wave. Using the vacuum impedance and frequencies in GHz the connection between the $\alpha$ parameter that leads to power control of the multiplication and the input plane wave power in mW is ${P}_{in}\left(\mathrm{mW}\right)=29.6\times {10}^{-10}{\nu }^{2}{\alpha }^{2}.$

Figures 4 and 5 give highlights of the optimization scheme. Figure 4 shows a direct comparison between calculated and measured harmonics by means of their relative emission power. Power control of the nonlinear effects giving rise to harmonic multiplication is driven by the parameter $\alpha =eEd/h\nu$, where e is the charge of the electron, *E* is the amplitude of the GHz input at frequency, $\nu$, *d* is the superlattice period, and *h* denotes Planck’s constant. Details of the underlying theory are given in Refs.^18–25^ and will not be repeated here. The actual power inside the superlattice is unknown and the best value for $\alpha$ is determined through simulated annealing. For the structure used in this work, the following parameters for simulation and experiment were used: input frequency $\nu =$ 178 GHz and $\alpha =eEd/h\nu =$ 19.6, consistently with our previous studies of samples with similar design.

Figure 5 shows a map that helps select the best level of input power to deliver maximum output for each harmonic. It also shows how an applied voltage can further control the output.

Giant control of the harmonics through a combination of input power and voltage has been predicted in good agreement with experiments recently^18^. We also predict a strong control by manipulating interface scattering asymmetry^23^, as well as a more complex combination of input power and biasing voltage controls^24^.

### Sampling technique and spectroscopic measurements

Patients’ characteristics are given in Table 2. Chemotherapy-naive patients without renal dysfunction, as confirmed by the results of the routine clinic tests (urine test, complete blood test, creatinine clearance), were enrolled. The characteristic toxicity of platinum derivatives (especially cisplatin) includes a high risk of kidney damage and renal failure (nephrotoxicity). The patients were notified about the necessary diet and the inadmissibility of alcohol drinking during the treatment. They were examined with routine blood tests, biochemical blood tests, and urine tests before and after chemotherapy.

**Table 2:** Patient characteristics with the level of creatinine in the blood before/after chemotherapy.

No	Gender, age	Diagnosis, stage	Chemotherapy type	Creatinine clearance mL/min/BSA
1	Female, 79	Ovarian cancer, iiic	Paclitaxel + cisplatin	88/95
2	Female, 68	Ovarian cancer, iiic	Cisplatin + etoposide	85/91
3	Female, 55	Ovarian cancer, iiic	Cisplatin	122/132
4	Female, 69	Ovarian cancer, iii	Paclitaxel + cisplatin	101/96
5	Male, 63	Pharyngeal cancer, iv	Cisplatin + 5-fu	82/94
6	Female, 72	Lung cancer, iiib	Carboplatin + paclitaxel	66/69

*BSA* body surface area.

The urine was sampled before chemotherapy and the day after, together with routine urine analysis according to guidelines of the Association of Russian Oncologists (AOR) to detect early urine changes. The urine samples of conditionally healthy volunteers were used as a control group.

The most commonly used drug in our study, cisplatin is a platinum-based alkylating agent. It is highly effective at treating many types of cancer. However, as pointed out in the introduction, these substances are nephrotoxic^7^. Our analysis allows us to detect metabolites that cannot be measured otherwise, but we did perform conventional clinical tests for a comparison. For example, we show the creatinine clearance levels before and a day after chemotherapy in Table 2. Creatinine clearance is reported as milliliters of creatinine per minute per body surface area (mL/min/BSA)^44^. Creatinine is a waste product that stems from the normal wear and tear on the muscles of the body. It is normal to have creatinine in the bloodstream, and the actual “normal value” can depend on age, muscular mass, and gender.

The creatinine clearance level in the blood plasma of all patients was within the normal range before and after cisplatin administration^44^. There were no changes in routine urine analyses.

**Figure 6:**
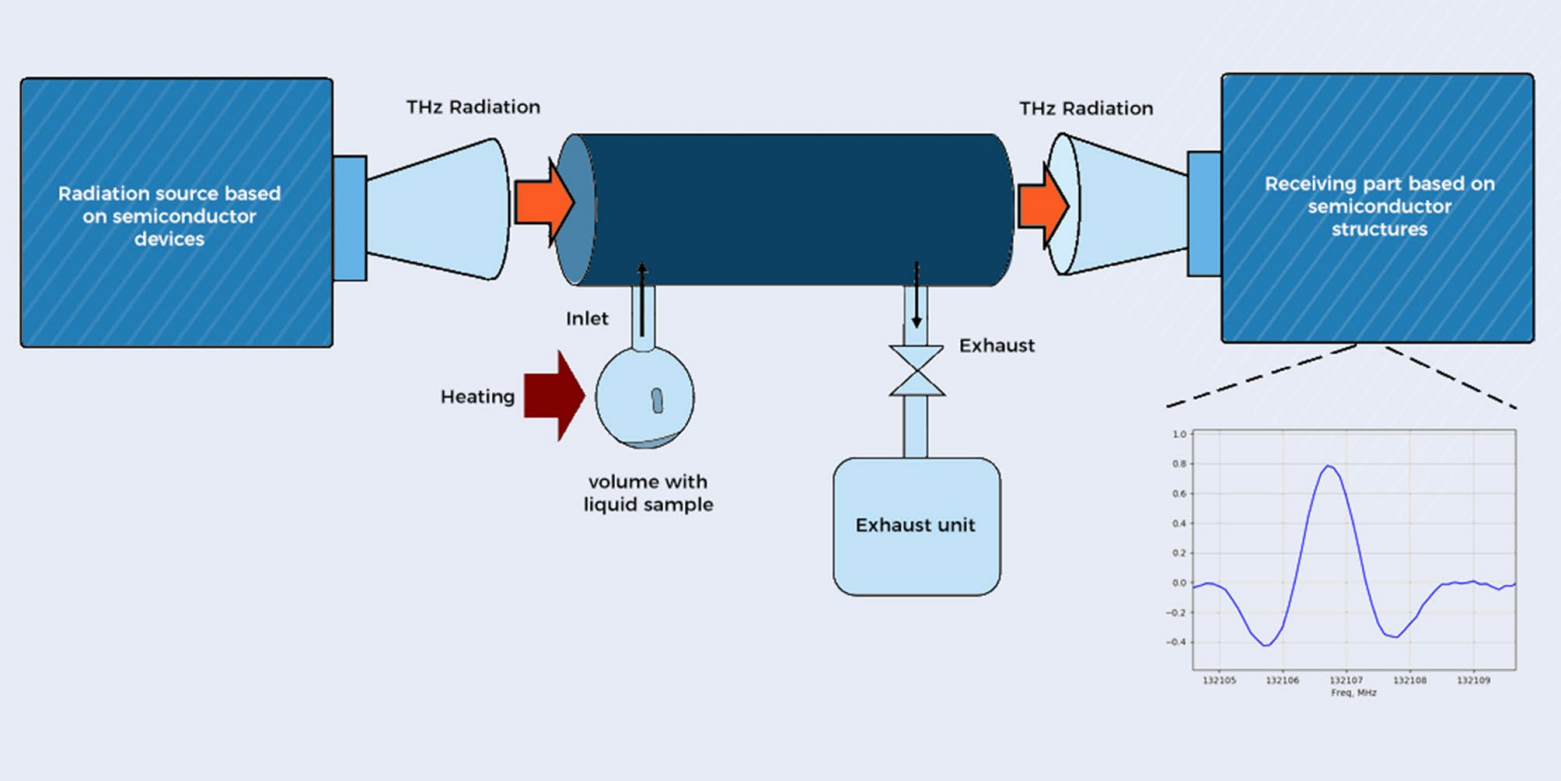
Diagram describing the experimental setup where the radiation source and the receiving part (detection) are based on semiconductor superlattice multipliers and mixers (SSLMs).

Urine samples of 1–2 ml were filled in a retort, and a cryogenic trap with liquid nitrogen was used for dehydration. First, the sample was frozen and then dehydrated to decrease the pressure of water vapors. A thin film or crystallized residue was formed in the retort, and volatile compounds from the residue passed in the measuring cell by natural evaporation and heating. The cell was evacuated to a preliminary pressure of 10^–4^ mbar. Absorption spectra of the vapors were measured with our high-resolution THz spectrometer, operating in phase switching mode^11^. Components of gas mixtures were identified using open-source spectral databases^31,32^. The study of urine vapor composition using THz spectroscopy allows us to detect all substances, appearing under thermal decomposition and having rotational spectra in the measured spectral range. The comparison of urine vapor composition before and after chemotherapy reveals the appearance of specific metabolites and details are given in the section “Results and discussion”. The experimental setup is depicted in Fig. 6.

Two types of spectrometers, based on either a BWO or a Gunn generator with frequency multiplying, were applied to study the urine composition of patients. The Gunn generator with frequency multiplying was used in the following ranges: 336–345 GHz (3rd harmonics), 560–575 GHz (5th harmonics) and 784–805 GHz (7th harmonics).

However, there are some difficulties in studying samples like urine in these frequency ranges, notably because the main content of products of thermal decomposition of urine consists of water (H_2_O), ammonia (NH3) including deuterated isotopologue (NH2D) and isocyanic acids (HNCO) and we need to avoid interference from their signature lines for conclusive diagnostics. To detect traces of nitrides, our spectrometer must have high sensitivity and resolution. Our phase-switching spectrometer applied to gas/vapor spectroscopy delivers an absorption coefficient sensitivity of 5 × 10^–10^ cm^−1^^45^ under standard measurement conditions, i.e. cell length of 1 m and averaging time of 1 s. For polar molecules such as NH_3_, CO, etc. and for operation in the 118–178 GHz range, this corresponds to detecting concentrations at a level of 1–100 ppb.

We present spectral lines for specific substances in the spectrum of urine. Some of these, such as HNCO, NH_3_ etc. appear in large concentration and in al samples. Our goal is to find nitriles that are in far smaller concentration mixed with these. Notably if they are not present before for this patient, or if they increase after chemotherapy, make them potential markers for early signs chemotherapy toxicity.

So the main issue is not the dynamic range determined by the detector, which per fabrication is 60 db, but rather if a given spectral line is present or not and we determine this by comparing the spectral position with the expected frequency in the databases^31,32^ and by confirming that the line has the expected lineshape.

The mechanisms that affect the spectral resolution are collisional and Doppler broadening. Collisional broadening is proportional to the pressure in measuring the cell, and it is about a hundred kHz − 1 MHz for most molecules.

Doppler broadening depends on frequency, temperature and mass and can be estimated by two standard approaches, namely $\Delta \nu$=1.17221 × 10^–6^ × ${\nu }_{r}$×(T/T_0_ × 28/M)^1/2^, where M is the mass of the absorber (in atomic mass units), T is temperature in K, T_0_ = 300 K and ${\nu }_{r}$ denotes the molecular rotational transition frequency^31,32^ or from $\Delta \nu$=3.581 × 10^–7^ × ${\nu }_{r}$×(T/M)^1/2^^46^. The difference between these relations is given by the coefficient of (28/300)^1/2^ ≈ 0.3055. For the substances investigated, the Doppler broadening does not exceed 200 kHz for room temperature or for 473 K (200 °C) in the ranges: 118–178 GHz, 450 GHz for 336–345 GHz (3rd harmonics), 750 GHz for 560–575 GHz (5th harmonics) and is about 1–1.5 MHz for 784–805 GHz (7th harmonics).

In the frequency range studied here, collisional and Doppler broadening are comparable, and they are distinguished by Lorentzian and Gaussian profiles, respectively. The actual experimental broadening results from a convolution of these, leading to a Voight line shape. In Figs. 1, 2 and 3 we show the quasi-second derivative of the spectra. This experimental absorption line is sharper than the original, while still centered at the same peak. This feature is a consequence of signal processing in our spectrometer. The broadening can thus be estimated as the difference between minima of the recorded absorption lines in the phase-switching mode^47^ characteristic of our sensor and is about 1–1.2 MHz in the 118–178 GHz range. The quasi-second derivative lineshape allows us to further distinguish a given spectral line in the case of mixtures of gases when it is difficult to resolve spectral peaks.

Note that we have detected a considerable number of spectral lines and showing all of them would make the paper unreadable and unfocused. Supplementary Tables S1–S3 and Table 1 summarize our findings in a far more efficient way. The figures with spectral lines deliver a few representative examples. Even though urine is heated up to about 200 °C, the vapors enter the measuring cell at room temperature and the absorption line shape depends on temperature and vapor pressure. As discussed above, the detected lines are quasi second derivatives from Voight line shapes. The signal-to-noise-ratio (SNR) in absorption lines can be resolved for SNR about 3 or lower and there are methods allowing to resolve lines for SNR close to 1. In our case the defining factor is not of good or bad SNR, but rather weather a line with specific form and linewidth is either present in the spectrum or not. See for example, Ref.^48^.

### Institutional review board and informed consent

The study was conducted according to the guidelines of the Declaration of Helsinki, and approved by the Institutional Review Board of Nizhny Novgorod Regional Oncology Hospital (protocol code 5, from 18.05.2015). Informed consent was obtained from all subjects involved in the study.

## Supplementary Information

Supplementary Information.

## Data Availability

Data discussed in this paper is given as Figures, Tables 1 and 2 of the main text and in Supplementary Tables S1–S3. The data files used to generate the Figures can be obtained from the authors upon reasonable request.

## References

[1] Clerici M (2013). Wavelength scaling of terahertz generation by gas ionization. Phys. Rev. Lett..

[2] Hartmann RR, Portion ME (2020). Guided modes and terahertz transitions for two-dimensional Dirac fermions in a smooth double-well potential. Phys. Rev. A.

[3] Villegas K, Kusmartsev F, Luo Y, Saviano I (2020). Optical transistor for an amplification of radiation in a broadband THz domain. Phys. Rev. Lett..

[4] Pereira, M. F. & Shulika, O. *Terahertz and Mid Infrared Radiation: Generation, Detection and Applications*. NATO Science for Peace and Security Series B: Physics and Biophysics. ISBN 978-94-007-0768-9. Springer Science Business Media B.V. 10.1007/978-94-007-0769-6 (2011).

[5] Dillon SS (2017). The 2017 terahertz science and technology roadmap. J. Phys. D.

[6] Wishart DS (2018). HMDB 4.0—The Human Metabolome Database for 2018. Nucleic Acids Res..

[7] Joint A (2018). Resting energy expenditure in the risk assessment of anticancer treatments. Clin. Nutr..

[8] Vaks VL (2012). Using the methods and facilities of no steady-state spectroscopy of the sub terahertz and terahertz frequency ranges for noninvasive medical diagnosis. J. Opt. Technol..

[9] https://ctep.cancer.gov/protocolDevelopment/electronic_applications/ctc.htm

[10] Vaks VL (2020). High resolution terahertz spectroscopy for analytical applications. Phys. Sup..

[11] Vaks V (2012). High-precise spectrometry of the terahertz frequency range: The methods, approaches and applications. J. Infared Mille Tirzah Waves.

[12] Easel H, Kimora R (2004). Submillimeter-wave InP Gunn devices. IEEE Trans. Microwave Theory Tech..

[13] Vaks VL (2021). High-resolution terahertz spectroscopy for investigation of energetic materials during their thermal decomposition. IEEE Trans. Terahertz Sci. Technol..

[14] Pavelyev DG, Skryl AS, Bakunov MI (2014). High-resolution broadband terahertz spectroscopy via electronic heterodyne detection of photonically generated terahertz frequency comb. Opt. Lett..

[15] Eisele H, Li L, Linfield EH (2018). High-performance GaAs/AlAs superlattice electronic devices in oscillators at frequencies 100–320 GHz. Appl. Phys. Lett..

[16] Gaifullin MB (2017). Microwave generation in synchronized semiconductor superlattices. Phys. Rev. Appl..

[17] Matharu S, Kusmartsev F, Balanov A (2013). High-frequency generation in two coupled semiconductor superlattices. Eur. Phys. J. Spec. Top..

[18] Pereira MF (2020). Giant controllable gigahertz to terahertz nonlinearities in superlattices. Sci. Rep..

[19] Pereira MF (2017). Theory and measurements of harmonic generation in semiconductor superlattices with applications in the 100 GHz to 1 THz range. Phys. Rev. B..

[20] Pereira MF, Anfertev VA, Zubelli JP, Vaks VL (2017). Terahertz generation by gigahertz multiplication in superlattices. J. Nanophoton..

[21] Apostolakis A, Pereira MF (2019). Controlling the harmonic conversion efficiency in semiconductor superlattices by interface roughness design. AIP Adv..

[22] Apostolakis A, Pereira MF (2019). Potential and limits of superlattice multipliers coupled to different input power sources. J. Nanophoton.

[23] Apostolakis A, Pereira MF (2020). Superlattice nonlinearities for Gigahertz-Terahertz generation in harmonic multipliers. Nanophotonics.

[24] Pereira MF, Apostolakis A (2021). Combined structural and voltage control of giant nonlinearities in semiconductor superlattices. Nanomaterials.

[25] Schevchenko Y, Apostolakis A, Pereira MF, Pereira MF, Apostolakis A (2021). Recent advances in superlattice frequency multipliers. Terahertz (THz), Mid Infrared (MIR) and Near Infrared (NIR) Technologies for Protection of Critical Infrastructures Against Explosives and CBRN. NATO Science for Peace and Security Series B: Physics and Biophysics.

[26] Razeghi M (2015). Quantum cascade lasers: from tool to product. Opt. Express.

[27] Valavanis A (2016). Diffuse-reflectance spectroscopy using a frequency-switchable terahertz quantum cascade laser. IEEE Trans. Terahertz Sci. Technol..

[28] Valavanis A (2015). Mechanically robust waveguide-integration and beam shaping of terahertz quantum cascade lasers. Electron. Lett..

[29] Wang F, Slivken S, Wu DH, Razeghi M (2020). Room temperature quantum cascade laser with ∼31% wall-plug efficiency. AIP Adv..

[30] https://www.nccn.org/professionals/physician_gls/pdf/head-and-neck.pdf; https://www.nccn.org/professionals/physician_gls/pdf/nscl.pdf; https://www.nccn.org/professionals/physician_gls/pdf/ovarian.pdf

[31] Pickett, H. M. *et al*. *Submillimeter, Millimeter, and Microwave Spectral Line Catalog. JPL Molecular Spectroscopy*. California Institute of Technology. http://spec.jpl.nasa.gov/ftp/pub/catalog/catform.html; 10.1364/ao.24.002235 18223871

[32] Endres CP, Schlemmer S, Schilke P, Stutzki J, Müller HSP (2016). The cologne database for molecular spectroscopy, CDMS, in the virtual atomic and molecular data centre. VAMDC. J. Mol. Spectrosc..

[33] Timerbayev NF, Safin RG, Sadrtdinov AR (2010). Modelling the process of cleaning the smoke gases produced at organic incineration. Vestnik KTU.

[34] Vaks V (2020). Application of THz fast frequency sweep spectrometer for investigation of chemical composition of blood. J. Infrared Millim. Terahz Waves.

[35] Lykina AA (2021). Terahertz high-resolution spectroscopy of thermal decomposition gas products of diabetic and non-diabetic blood plasma and kidney tissue pellets. J. Biomed. Opt..

[36] Von Jakubke, H.‐D. & Jeschkeit, H. Aminosäuren, Peptide, Proteine 505 S(Verlag Chemie, Weinheim, 1982).

[37] Townsend DM, Deng M, Zhang L (2003). Metabolism of Cisplatin to a nephrotoxin in proximal tubule cells. J. Am. Soc. Nephrol..

[38] Kuhlmann MK, Burkhardt G, Kohler H (1997). Insights into potential cellular mechanisms of cisplatin nephrotoxicity and their clinical application. Nephrol. Dial. Transplant..

[39] Townsend DM, Hanigan MH (2002). Inhibition of gamma-glutamyl transpeptidase or cysteine S-conjugate beta-lyase activity blocks the nephrotoxicity of cisplatin in mice. J. Pharmacol. Exp. Ther..

[40] Yablokov VA, Vasina YA, Zelyaev IA, Mitrofanova SV (2009). Kinetics of thermal decomposition of sulfur-containing amino acids. Russ. J. Gen. Chem..

[41] Al-Shukri S, Goloschapov E, Emanuel Yu, Gobachev M (2012). Tamm-Horsfall protein—potential marker of early stages urolit hiasis and stone recurrence. Urologicheskie Vedomosti.

[42] Papayan AV, Lisovaya NA (2002). Role of Tamm-Horsfall protein in renal diseases. Nephrology.

[43] Wu T-H, Li K-J, Yu C-L, Tsai CY (2018). Tamm-Horsfall protein is a potent immunomodulatory molecule and a dis-ease biomarker in the urinary system. Molecules.

[44] https://www.mayoclinic.org/tests-procedures/creatinine-test/about/pac-20384646

[45] Brailovsky AB, Khodos VV, Vaks VL (1999). Millimeter range spectrometer with phase switching-novel method for reaching of the top sensitivity. Int. J. Infrared Millim. Waves.

[46] Townes CH, Schawlow AL (1955). Microwave Spectroscopy.

[47] Gordy W, Cook RL (1984). Microwave Molecular Spectra.

[48] Rothbart N (2022). Millimeter-Wave Gas Spectroscopy For Breath analysis of COPD patients in comparison to GC-MS. J. Breath Res..

