# Author Correction: Sensing nitriles with THz spectroscopy of urine vapours from cancers patients subject to chemotherapy

**DOI:** 10.1038/s41598-023-33756-1

**Published:** 2023-04-21

**Authors:** Vladimir Vaks, Vladimir Anfertev, Maria Chernyaeva, Elena Domracheva, Anton Yablokov, Anna Maslennikova, Alla Zhelesnyak, Alexei Baranov, Yuliia Schevchenko, Mauro Fernandes Pereira

**Affiliations:** 1 Institute for Physics of Microstructures, Nizhny Novgorod, Russia 603950; 2 Lobachevsky State University, Nizhny Novgorod, Russia 603950; 3 Privolzhsky Research Medical University, Nizhny Novgorod, Russia 603005; 4 Nizhny Novgorod Regional Oncology Hospital, Nizhny Novgorod, Russia 603000; 5 Institute of Electronics and Systems (IES), University of Montpellier, UMR5214 CNRS/Université, Montpellier 2, 34095 Montpellier, France; 6 Department of Physics, Khalifa University of Science and Technology, 127788 Abu Dhabi, United Arab Emirates; 7 Institute of Physics, Czech Academy of Sciences, 18221 Prague, Czech Republic

Correction to: *Scientific Reports* https://doi.org/10.1038/s41598-022-22783-z, published online 27 October 2022

In the original version of this Article, Affiliations 6 and 7 were not listed in the correct order. The correct affiliations are listed below:

Affiliation 6:

Department of Physics, Khalifa University of Science and Technology, 127788 Abu Dhabi, United Arab Emirates

Affiliation 7:

Institute of Physics, Czech Academy of Sciences, 18221 Prague, Czech Republic

The original Article has been corrected.

