# Supplementary material for: Sensing nitriles with THz spectroscopy of urine vapours from cancers patients subject to chemotherapy
Source: Sci Rep. 2022 Oct 27;12:18117. doi: 10.1038/s41598-022-22783-z (PMC9613899; doi:10.1038/s41598-022-22783-z)
Supplement: Supplementary file 1 — Supplementary Information. [file 41598_2022_22783_MOESM1_ESM.docx]

**Supplementary Material**

**Sensing nitriles with THz spectroscopy of urine vapours from cancers patients subject to chemotherapy**

**Vladimir Vaks^1,2^, Vladimir Anfertev^1^, Maria Chernyaeva^1,2^, Elena Domracheva^1^, Anton Yablokov^1^, Anna Maslennikova^2,3^, Alla Zhelesnyak^4^, Alexei Baranov^5^, Yuliia Schevchenko^6^ and Mauro Fernandes Pereira^6,7*^**

^1^Institute for Physics of Microstructures, Nizhny Novgorod, 603950, Russia

^2^Lobachevsky State University, Nizhny Novgorod, 603950, Russia

^3^Privolzhsky Research Medical University, Nizhny Novgorod, 603005, Russia

^4^Nizhny Novgorod Regional Oncology Hospital, Nizhny Novgorod 603000, Russia

^5^Institute of Electronics and Systems (IES), University of Montpellier, UMR5214 CNRS/Université Montpellier 2, 34095 Montpellier, France

^6^Institute of Physics, Czech Academy of Sciences, 18221 Prague, Czech Republic

^7^Department of Physics, Khalifa University of Science and Technology, Abu Dhabi 127788, UAE

*mauro.pereira@ku.ac.ae

Tables S1 to S3 illustrate how we identify the substances found in the urine vapor by comparing experimental lines with corresponding data found in the JPL and Köln databases [31, 32]. They combine representative examples of substances found in all samples and some from the sample of patient 2 after chemotherapy including relevant nitriles. The common logarithm of the integral intensity (Lg I) characterizes the absorption intensity measured at 300 K (I is in nm^2^ MHz) and the quantum numbers are given in the following order [31, 32] in the Tables: J (or N); Ka and Kc (or ± K); v; F1 . . . F for the upper state followed by the corresponding lower state numbers after the arrow (←). Here, J is the total rotational angular momentum including electron spin and is N the total rotational angular momentum excluding electron and nuclear spins; Ka and Kc are the projections of N onto the A and C inertial axes, respectively. In the case of symmetric top molecules, they are replaced by K, which define the parity; v (or vt, v30 etc.) is a state number, specifying different vibrational or electronic states. In a few relevant cases, the vibrational state is indicated with the molecular nomenclature, e.g., for NH_3_; F1… F designates spin quanta for the upper and lower states. Either the first three quantum numbers are given, or if the transition is between vibrational states, it is designated by letters v, vt, and v30 as in databases [31, 32].

**Table S1.** Spectral data of typical substances measured in all urine samples in the 784-805 GHz range.

| **Substance** | **Experimental central frequency, GHz** | **Frequency from database, GHz** | **Lg I, I [nm^2^*MHz]**  **from database** | **Quantum numbers** |
| --- | --- | --- | --- | --- |
| NH3-v2  ammonia | 790.818 | 790.8175468 | -4.4654 | 10 6 3 ← 10 -6 2 |
|  | 791.531 | 791.5308740 | -4.4031 | 8 3 3 ← 8 -3 2 |
| NH2D  deuterated ammonia | 785.598 | 785.5972255  785.5978379  785.5978478  785.5979627  785.5979746  785.5984603  785.5987119 | -2.6947  -4.1490  -4.1490  -4.1503  -4.1503  -2.6049  -2.7701 | 5 3 2 0 5 ← 5 2 4 1 5  5 3 2 0 5 ← 5 2 4 1 6  5 3 2 0 6 ← 5 2 4 1 5  5 3 2 0 5 ← 5 2 4 1 4  5 3 2 0 4 ← 5 2 4 1 5  5 3 2 0 6 ← 5 2 4 1 6  5 3 2 0 4 ← 5 2 4 1 4 |
|  | 785.702 | 785.7022888 | -2.0532 | 5 4 2 1 ← 5 3 2 0 |
|  | 788.746 | 788.7457634 | -4.8462 | 11 4 7 1 ← 11 4 8 1 |
|  | 790.190 | 790.1899425 | -5.2159 | 11 4 7 0 ← 11 4 8 0 |
|  | 790.832 | 790.8319020  790.8320268  790.8326392 | -5.9868  -5.9855  -4.5312 | 5 2 4 1 4 ← 5 0 5 1 5  5 2 4 1 6 ← 5 0 5 1 5  5 2 4 1 5 ← 5 0 5 1 5 |
|  | 790.834 | 790.8339404  790.8342055  790.8345529  790.8349427 | -4.4414  -4.6066  -5.9855  -5.9868 | 5 2 4 1 6 ← 5 0 5 1 6  5 2 4 1 4 ← 5 0 5 1 4  5 2 4 1 5 ← 5 0 5 1 6  5 2 4 1 5 ← 5 0 5 1 4 |
|  | 791.716 | 791.7145210  791.7146463  791.7152615  791.7165614  791.7168262  791.7171765  791.7175666 | -6.3617  -6.3604  -4.9061  -4.8163  -4.9815  -6.3604  -6.3617 | 5 2 4 0 4 ← 5 0 5 0 5  5 2 4 0 6 ← 5 0 5 0 5  5 2 4 0 5 ← 5 0 5 0 5  5 2 4 0 6 ← 5 0 5 0 6  5 2 4 0 4 ← 5 0 5 0 4  5 2 4 0 5 ← 5 0 5 0 6  5 2 4 0 5 ← 5 0 5 0 4 |
|  | 792.605 | 792.6059688 | -4.8687 | 9 3 6 1 ← 9 3 7 1 |
|  | 792.965 | 793.9659050 | -4.2762 | 9 3 6 0 ← 9 3 7 0 |
|  | 795.593 | 795.5929855 | -3.7248 | 12 6 7 0 ← 12 5 7 1 |
| HNCO  isocyanic acid | 787.378 | 787.3780619  787.3780614  787.3780619  787.3780614  787.3780651 | -4.5746  -4.5505  -4.5746  -4.5505  -4.5626 | 36 6 31 35 ← 35 6 30 34  36 6 31 37 ← 35 6 30 36  36 6 30 35 ← 35 6 29 34  36 6 30 37 ← 35 6 29 36  36 6 30 36 ← 35 6 29 35 |
|  | 787.704 | 787.7030552  787.7039125  787.7039130  787.7039131  787.7047471 | -5.4703  -2.3460  -2.3580  -2.3701  -5.4703 | 36 1 36 35 ← 35 1 3535  36 1 36 37 ← 35 1 35 36  36 1 36 36 ← 35 1 35 35  36 1 36 35 ← 35 1 35 34  36 1 36 36 ← 35 1 35 36 |
|  | 789.221 | 789.2204035  789.2204035  789.2211149  789.2211149  789.2211154  789.2211154  789.2211176  789.2211176  789.2218097  789.2218097 | -6.9822  -6.9822  -3.8579  -3.8579  -3.8820  -3.8820  -3.8699  -3.8699  -6.9822  -6.9822 | 36 5 31 35 ← 35 5 30 35  36 5 32 35 ← 35 5 31 35  36 5 32 37 ← 35 5 31 36  36 5 31 37 ← 35 5 30 36  36 5 32 35 ← 35 5 31 34  36 5 31 35 ← 35 5 30 34  36 5 32 36 ← 35 5 31 35  36 5 31 36 ← 35 5 30 35  36 5 31 36 ← 35 5 30 36  36 5 32 36 ← 35 5 31 36 |
|  | 789.780 | 789.7792814  789.7792828  789.7800089  789.7800094  789.7800107  789.7800102  789.7800108  789.7800120  789.7807184  789.7807198 | -6.4154  -6.4154  -3.2910  -3.3152  -3.3031  -3.2910  -3.3152  -3.3031  -6.4154  -6.4154 | 36 4 33 35 ← 35 4 32 35  36 4 32 35 ← 35 4 31 35  36 4 33 37 ← 35 4 32 36  36 4 33 35 ← 35 4 32 34  36 4 33 36 ← 35 4 32 35  36 4 32 37 ← 35 4 31 36  36 4 32 35 ← 35 4 31 34  36 4 32 36 ← 35 4 31 35  36 4 33 36 ← 35 4 32 36  36 4 32 36 ← 35 4 31 36 |
|  | 790.182 | 790.1812139  790.1819536  790.1819542  790.1819547  790.1820005  790.1826744  790.1827402  790.1827407  790.1827413  790.1834610 | -5.9745  -2.8502  -2.8743  -2.8623  -5.9745  -5.9745  -2.8502  -2.8743  -2.8623  -5.9745 | 36 3 34 35 ← 35 3 33 35  36 3 34 37 ← 35 3 33 36  36 3 34 35 ← 35 3 33 34  36 3 34 36 ← 35 3 33 35  36 3 33 35 ← 35 3 32 35  36 3 34 36 ← 35 3 33 36  36 3 33 37 ← 35 3 32 36  36 3 33 35 ← 35 3 32 34  36 3 33 36 ← 35 3 32 35  36 3 33 36 ← 35 3 32 36 |
|  | 790.416 | 790415.4123  790416.1627  790416.1633  790416.1634  790416.8935 | -5.6597  -2.5353  -2.5595  -2.5474  -5.6597 | 36 2 35 35 ← 35 2 34 35  36 2 35 37 ← 35 2 34 36  36 2 35 35 ← 35 2 34 34  36 2 35 36 ← 35 2 34 35  36 2 35 36 ← 35 2 34 36 |
|  | 790.518 | 790.5174192  790.5181814  790.5181820  790.5181820  790.5189237 | -5.4078  -2.2835  -2.3076  -2.2956  -5.4078 | 36 0 36 35 ← 35 0 35 35  36 0 36 37 ← 35 0 35 36  36 0 36 35 ← 35 0 35 34  36 0 36 36 ← 35 0 35 35  36 0 36 36 ← 35 0 35 36 |
|  | 790.577 | 790.5759375  790.5766822  790.5766826  790.5766828  790.5774072 | -5.6596  -2.5353  -2.5473  -2.5594  -5.6596 | 36 2 34 35 ← 35 2 33 35  36 2 34 37 ← 35 2 33 36  36 2 34 36 ← 35 2 33 35  36 2 34 35 ← 35 2 33 34  36 2 34 36 ← 35 2 33 36 |
|  | 790.995 | 790.9943285  790.9949864  790.9949960  790.9950176  790.9951580  790.9958254 | -4.1575  -2.8687  -2.7772  -2.6870  -6.1531  -4.1574 | 4 1 4 5 ← 5 0 5 5  4 1 4 3 ← 5 0 5 4  4 1 4 4 ← 5 0 5 5  4 1 4 5 ← 5 0 5 6  4 1 4 5 ← 5 0 5 4  4 1 4 4 ← 5 0 5 4 |
|  | 793.451 | 793.4507105  793.4513657  793.4513661  793.4513661  793.4520035 | -5.4712  -2.3469  -2.3710  -2.3589  -5.4712 | 36 1 35 35 ← 35 1 34 35  36 1 35 37 ← 35 1 34 36  36 1 35 35 ← 35 1 34 34  36 1 35 36 ← 35 1 34 35  36 1 35 36 ← 35 1 34 36 |

**Table S2.** Spectral data of typical substances measured in the urine sample of patient 2 after chemotherapy.

| **Substance** | **Experimental central frequency, GHz** | **Frequency from database, GHz** | **Lg I, I [nm^2^*MHz]**  **from database** | **Quantum numbers** |
| --- | --- | --- | --- | --- |
| HDO  semiheavy water | 143.7271 | 143.72721 | -3.6215 | 4 2 2 ← 4 2 3 |
| NH3  ammonia | 140.1412 | 140.1418067 | -5.0383 | 2 1 2 ← 1 1 3, v2 |
| HNCO  isocyanic acid | 134.4818  138.9100 | 134.4816614  134481.6650  134481.7571  138.9097510  138.9097510  138.9097510 | -4.2090  -4.1827  -4.1959  -4.6760  -4.6661  -4.6859 | 32 1 32 31← 33 0 33 32  32 1 32 33 ← 33 0 33 34  32 1 32 32 ← 33 0 33 33  44 0 44 44 ← 43 1 43 43  44 0 44 45 ← 43 1 43 44  44 0 44 43 ← 43 1 43 42 |
| CH3COOH  acetic acid | 146.8891 | 146.8892045 | -5.5563 | 16 12 5 2 2 ← 16 11 6 2 1 vt=2 |
| CH3CHO  acetaldehyde | 147.1922 | 147.192134 | -5.7062 | 29 4 26 3 ← 28 5 23 3 |
| a-C2H5SH  ethanethiol | 148.0133 | 148.0132725  148.0132725 | -5.5024  -5.5024 | 45 14 31 ← 46 13 34  45 14 32 ← 46 13 33 |

**Table S3.** Spectral data measured in the urine sample of patient 2 after chemotherapy (Nitriles).

| **Substance** | **Experimental central frequency, GHz** | **Frequency from database, GHz** | **Lg I, I [nm^2^*MHz]**  **from database** | **Quantum numbers** |
| --- | --- | --- | --- | --- |
| C2H3CN  acrylonitrile | 147.2065 | 147.2066281  147206.6335  147206.6536 | -5.7849  -5.7365  -5.7607 | 18 0 18 2 17 ← 17 1 17 2 16  18 0 18 2 19 ← 17 1 17 2 18  18 0 18 2 18 ← 17 1 17 2 17 |
| C2H5CN  propionitrile | 149.1042 | 149.104309 | -5.4684 | 50 4 46 ← 50 4 47 |
| i-C3H7CN  isobutyronitrile | 145.9481  120.4366 | 145.9479272  145.9478961  120.4364588 | -5.0754  -5.0754  -3.7040 | 38 6 33 ← 38 4 34(v30=1)  38 5 33 ← 38 5 34 (v30=1),  17 7 11 ← 16 7 10 |
| AA-n-C4H9CN  n-butyl-cyanide | 148.5347 | 148.5346238 | -4.9589 | 42 10 32← 43 9 35  42 10 33 ← 43 9 34 |
| C2H5CHCNCH3  butyronitrile | 132.5015  145.6832  168.350.0 | 132.5014850  145.6833846  145.6833846  168.3498414  168.349.8414  168.349.8414  168.349.8414 | -6.3800  -4.9041  -4.9041  -4.4983  -5.0689  -5.0689  -4.4983 | 120 66 55 ← 120 65 56 v = 0  64 54 10←64 53 11 v=0  64 54 11 ← 64 53 12 v=0  61 9 52 ← 61 8 53 v = 0  61 9 52 ← 61 9 53 v = 0  61 10 52 ← 61 8 53 v = 0  61 10 52 ← 61 9 53 v = 0 |
| c-C6H5CN  benzonitrile | 132.7130  173.8551 | 132.7130395  173.8547222 | -4.6476  -3.3369 | 48 42 6 ← 47 42 5  48 42 7 ← 47 42 6  65 659← 64 658 |
